# TelomereHunter2: improved *in silico* telomere analysis software for precision oncology and single-cell studies

**DOI:** 10.1093/bioadv/vbag187

**Published:** 2026-06-30

**Authors:** Ferdinand Popp, Nicola Biondi, Urška Pogorevčnik, Nicholas Abad, Chen Hong, Yoann Pageaud, Benedikt Brors, Lars Feuerbach

**Affiliations:** Division Applied Bioinformatics, German Cancer Research Center (DKFZ), Heidelberg, 69120, Germany; Faculty of Biosciences, Heidelberg University, Heidelberg, 69120, Germany; Division Applied Bioinformatics, German Cancer Research Center (DKFZ), Heidelberg, 69120, Germany; Faculty of Biosciences, Heidelberg University, Heidelberg, 69120, Germany; Division Applied Bioinformatics, German Cancer Research Center (DKFZ), Heidelberg, 69120, Germany; Division Applied Bioinformatics, German Cancer Research Center (DKFZ), Heidelberg, 69120, Germany; Faculty of Biosciences, Heidelberg University, Heidelberg, 69120, Germany; Division Applied Bioinformatics, German Cancer Research Center (DKFZ), Heidelberg, 69120, Germany; Division Applied Bioinformatics, German Cancer Research Center (DKFZ), Heidelberg, 69120, Germany; Faculty of Biosciences, Heidelberg University, Heidelberg, 69120, Germany; Division Applied Bioinformatics, German Cancer Research Center (DKFZ), Heidelberg, 69120, Germany; Faculty of Biosciences, Heidelberg University, Heidelberg, 69120, Germany; National Center for Tumor Diseases (NCT), Heidelberg, 69120, Germany; German Cancer Consortium (DKTK), Heidelberg, 69120, Germany; Medical Faculty Heidelberg, Heidelberg University, Heidelberg, 69120, Germany; Division Applied Bioinformatics, German Cancer Research Center (DKFZ), Heidelberg, 69120, Germany

**Keywords:** Telomeres, Bioinformatics, Computational Genomics, Sequence Analysis, Next-Generation Sequencing

## Abstract

**Motivation:**

Telomere biology plays a critical role in multiple biological processes including carcinogenesis, aging, and genome stability. With increasing availability of DNA-sequence datasets, telomere length and composition are more frequently directly inferred *in silico*. The TelomereHunter software is used in genome research and precision oncology to study telomere maintenance mechanisms from routine sequencing data. However, bioinformatics tools face constant challenges such as increasing the number and size of genomic datasets, novel file formats and deprecating software components.

**Results:**

We developed TelomereHunter2 (TH2) to create a sustainable framework for telomere analysis. By containerizing our software and improving the runtime by up to 74%, we simplify the integration of TH2 into diverse precision oncology workflows and computational environments. We also extended TH2 to support non-human genomes and single-cell sequencing approaches, broadening its applications across species and methodologies. We demonstrate TH2 improvements on a pilot dataset.

**Availability and implementation:**

TelomereHunter2 is an open-source Python package released under the GPL-3.0 license. It is distributed via PyPI and the source code, documentation, and wiki are available at: https://github.com/ferdinand-popp/TelomereHunter2.

## 1 Introduction

Telomeres are the protective ends of linear chromosomes that prevent genomic instability. They shorten during each cell division, until senescence is triggered at a critical length (M1 checkpoint/Hayflick limit). Cancer cells overcome this limitation through telomere maintenance mechanisms (TMMs): either by reactivating the telomerase enzyme (TERT) or through alternative lengthening of telomeres (ALT) (Bryan *et al*. 1997).

The TelomereHunter software is a bioinformatics tool to support the digital diagnosis of both TMM activity and type from genome sequencing data (Feuerbach *et al*. 2019). For instance, in neuroblastoma, TMM status holds significant prognostic value and is used to stratify patients into distinct prognostic groups, aiding in risk assessment and treatment planning discussions (Hartlieb *et al*. 2021). It is routinely used in large-scale personalized oncology programs, such as MASTER and INFORM (Jäger 2022). The generated output features such as telomere content and telomere variant repeats (TVRs) improve machine learning models for TMM type prediction and disorders (Sieverling *et al*. 2020, Sanchez *et al*. 2024). Additionally, targeted therapies such as the telomerase inhibitor Imetelstat, which was recently FDA-approved for myelodysplastic syndromes, are being explored for their potential to improve patient outcomes (Platzbecker *et al*. 2024). With the onset of telomere-based therapies, tools to characterize TMMs *in silico* are becoming increasingly important for advancing precision medicine. Beyond cancer, telomeres are critical markers of aging and immune function, with lengths declining throughout life despite hematopoietic telomerase activity (Blackburn *et al*. 2015). Large-scale studies, such as the UK Biobank analysis encompassing over 450 000 genomes, have revealed new insights into telomere biology across diverse research fields, including cancer, aging, and immune system regulation (Burren *et al*. 2024).

Thus, the rapid growth of biomedical genome datasets necessitates efficient tools to handle the increasing scale and complexity of these analyses. Challenges include growing dataset sizes, novel file formats (e.g. CRAM), deprecating software components, and runtime optimization. These gaps motivated the development of TelomereHunter2 (TH2), a reimplementation and expansion of the widely used TelomereHunter.

## 2 Results

### 2.1 General software challenges

**Figure 1:**
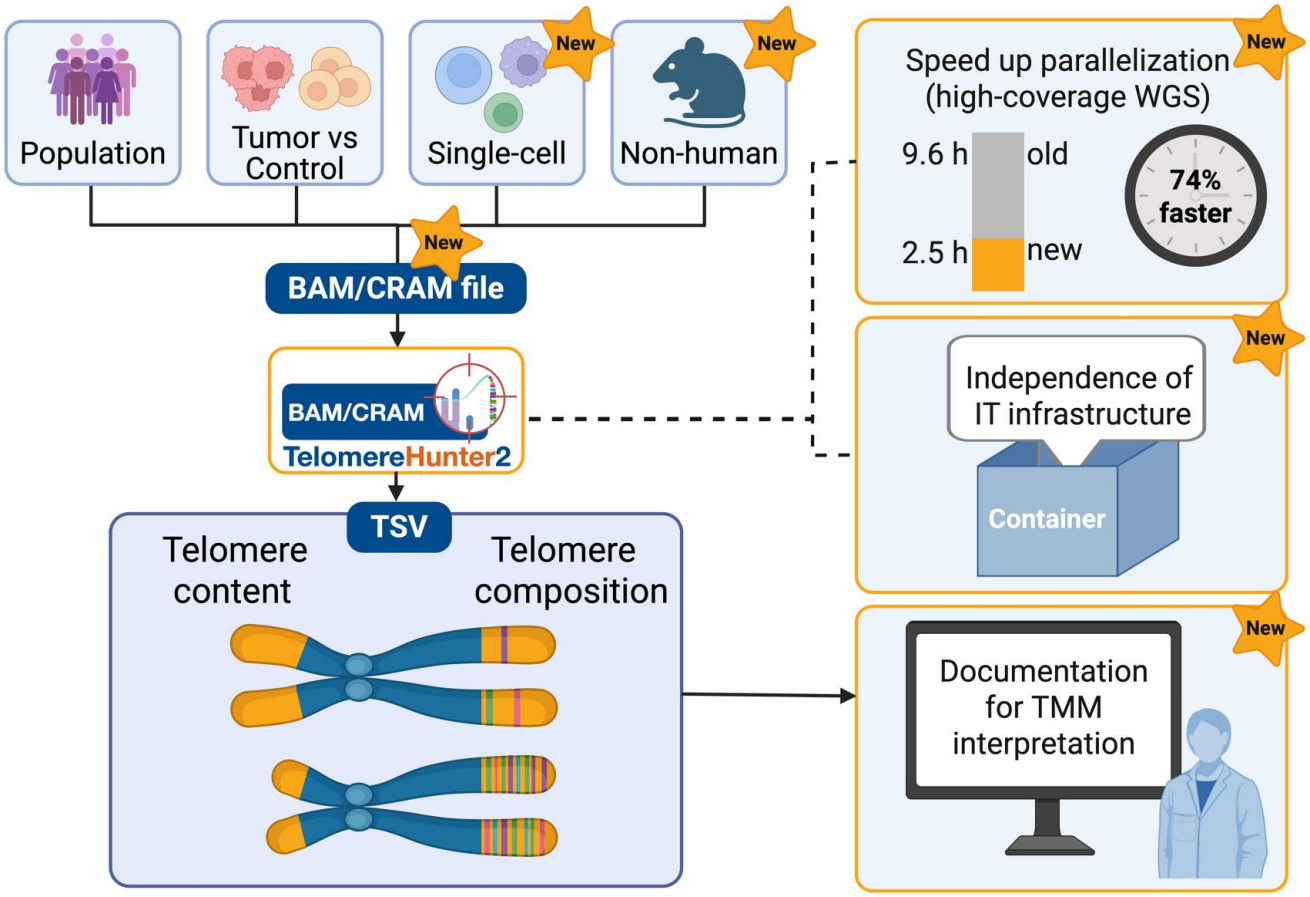
TelomereHunter2 schematic workflow and improvements compared to TelomereHunter. Speed up by parallelization depicted for ∼141× coverage of whole-genome sequencing files. Created in BioRender: Popp, F. (2026) https://BioRender.com/zl3bntv. TMM: telomere maintenance mechanism.

TH2 addresses these software lifecycle challenges with a complete Python3 reimplementation (Fig. 1). This eliminates prior dependencies on Python2, R, and external Samtools installations, significantly simplifying setup. In addition to the BAM format, it directly supports CRAM format which avoids time-consuming format remapping. Further, it supports hg19, hg38, T2T, and customized reference genomes. Comprehensive documentation and improved error handling have been added to the repository.

### 2.2 Clinical application and benchmarking

Adapting the software in the precision oncology field adds new requirements, such as meeting strict clinical deadlines and deployment in different hospital IT systems. TH2 solves these challenges through several improvements:

First, containerization with Docker and Apptainer allows TH2 to run consistently across various computing environments without complex setup. Second, to improve runtime, we optimized key algorithmic steps and improved parallelization. Benchmarking on 8 high-coverage WGS (hc-WGS, ∼141× coverage) and 20 low-coverage WGS (lc-WGS, ∼4×) files demonstrated substantial performance improvements. Our optimization resulted in up to 74% runtime reduction (3.8× speedup) for combined hc-WGS files when compared to TH1 with standard settings (Table S1). This decreased wall time from approximately 9–10 hours to 2.5 hours. Even with running tumor and control analysis in parallel for TelomereHunter (TH1_pl; average 6 hours), TH2 demonstrated superior performance. While TH2 fully supports both BAM and CRAM format inputs, the current version shows no major speed drawback for CRAM of medium compression level of over 50% size reduction (hc-WGS: BAM average 2.4 hours; CRAM average 2.6 hours). TH2 uses more main memory to facilitate the speedup (TH1 BAM 250 MB; TH2 BAM 650 MB; TH2 CRAM 990 MB). For lc-WGS, wall time decreased by 69%, from about 33 mins to 10 mins on average. The telomere content values of TH1 and TH2 highly correlate: Pearson r = 1.000 (*P* = 4.63e-56), Spearman r = 0.999 (*P* = 5.01e-40) (Fig. S1; Table S1). This implies that the extensive validation that was conducted for TH1 transfers to TH2 results. All benchmark tests were conducted on an IBM LSF cluster system with AMD EPYC 740 224-Core Processor on CentOS Linux 7 utilizing 8 cores, Python 3.10, and 10 GB RAM allocation for TelomereHunter2 version 1.0.0. and TelomereHunter version 1.1.

### 2.3 Non-human genome applications

TH2 introduces support for non-human genomes, enabling telomere analysis across species. By customizing cytoband reference, we successfully analyzed mouse (n = 35) and dog (n = 22) WGS genomes and observed differences in telomere content depending on breed and subspecies (Figs. S2 and S3). This demonstrates TH2's applicability in comparative genomics.

### 2.4 Single-cell ATAC analysis

In addition to its main bulk analysis mode, TH2 has a single-cell analysis mode. For each cell barcode, it provides individual telomere content and TVR measurements. To include only cells with sufficient coverage and ensure robust results, the single-cell mode requires an additional parameter of minimum reads per barcode. Simulations to select this at 30 000 reads per barcode and a showcase analysis of scATAC data with TelomereHunter2 are present in the paper from Engel *et al*. (2026). Exemplary results for a scATAC human PBMC dataset analysed on the above-mentioned compute cluster are appended in Fig. S4 and the analysis documented in the repository.

## 3 Discussion

TelomereHunter2 (TH2) provides a robust and sustainable framework for telomere analysis through its Python3-based reimplementation. The tool addresses critical bioinformatics challenges, including the need for faster processing of large datasets and direct support for CRAM format, eliminating the need for time-consuming format conversions. These improvements reduce computational overhead, enabling cost-effective analysis of high-coverage genomes and make TH2 particularly suitable for large-scale cohort studies and clinical applications. TelomereHunter was primarily developed for the analysis of medium sized cohorts of matched tumor and control genomes. TelomereHunter2 additionally supports the analysis of non-human genomes. Further, it enables *in silico* telomere analysis at single-cell resolution, thus broadening its applicability to diverse research areas, including comparative genomics, aging, and studies of the tumor microenvironment and the immune system.

Our tool provides essential preprocessing of biological signals from the non-coding genome, making complex information on telomere content and composition available as features for machine learning and AI applications. Hence, by lowering the access barrier to *in silico* telomere analysis, TH2 facilitates the use of telomere characteristics as biomarkers in oncology, immunology, and aging research. In times of agentic coding, it is important to have peer-reviewed stable reference tools.

We open-sourced our repository with a GPL-3.0 license under: https://github.com/ferdinand-popp/telomerehunter2 with additional information under https://www.dkfz.de/angewandte-bioinformatik/telomerehunter.

## Supplementary Material

vbag187_Supplementary_Data

## Data Availability

TelomereHunter2 is an open-source Python package released under the GPL-3.0 license. It is distributed via PyPI and the source code, documentation, and wiki (including tutorials and videos) are available at: https://github.com/ferdinand-popp/TelomereHunter2. Container files are also in the repository and on Docker Hub.
