# Supplementary material for: TelomereHunter2: improved in silico telomere analysis software for precision oncology and single-cell studies
Source: Bioinform Adv. 2026 Jun 30;6(1):vbag187. doi: 10.1093/bioadv/vbag187 (PMC13361702; doi:10.1093/bioadv/vbag187)
Supplement: vbag187_Supplementary_Data [file vbag187_supplementary_data.docx]

## Supplementary Material

| **Group** | **Configuration** | **TH1_walltime (min)** | **TH1_memory (MB)** | **TH1_pl_walltime (min)** | **TH1_pl_memory (MB)** | **TH2_walltime (min)** | **TH2_memory (MB)** | **TH1_vs_TH2_walltime (%)** | **TH1_tel_content log2(T/C)** | **TH2_tel_content log2(T/C)** |
| --- | --- | --- | --- | --- | --- | --- | --- | --- | --- | --- |
| **hcWGS BAM** | Patient_1 | 544.5 | 135 | 306.6 | 171 | 120.4 | 605 | -77.9 | -0.6509 | -0.6509 |
| **hcWGS BAM** | Patient_2 | 736.8 | 167 | 467.8 | 166 | 191.2 | 609 | -74.0 | -0.4899 | -0.4899 |
| **hcWGS BAM** | Patient_3 | 438.7 | 148 | 292.0 | 163 | 194.2 | 603 | -55.7 | -0.1763 | -0.1763 |
| **hcWGS BAM** | Patient_4 | 595.4 | 353 | 395.3 | 349 | 170.8 | 610 | -71.3 | 1.2516 | 1.2516 |
| **hcWGS BAM** | Patient_5 | 652.3 | 148 | 313.1 | 148 | 94.2 | 607 | -85.6 | -549 | -549 |
| **hcWGS BAM** | Patient_6 | 583.8 | 245 | 407.4 | 245 | 207.6 | 604 | -64.5 | 0.6747 | 0.6747 |
| **hcWGS BAM** | Patient_7 | 480.2 | 206 | 334.0 | 206 | 150.5 | 606 | -68.7 | -271 | -271 |
| **hcWGS BAM** | Patient_8 | 491.8 | 187 | 372.5 | 187 | 111.5 | 605 | -77.3 | 294 | 294 |
| **hcWGS BAM** | avg. hcWGS BAM | 565.4 | 199 | 361.1 | 204 | 155.0 | 606 | -72.6 |  |  |
| **hcWGS CRAM** | Patient_1 | 827.4 | 212 | 350.0 | 274 | 129.9 | 940 | -84.3 | -0.6509 | -0.6509 |
| **hcWGS CRAM** | Patient_2 | 788.7 | 247 | 361.6 | 247 | 168.2 | 995 | -78.7 | -0.4899 | -0.4899 |
| **hcWGS CRAM** | Patient_3 | 458.7 | 162 | 301.7 | 185 | 116.5 | 925 | -74.6 | -0.1763 | -0.1763 |
| **hcWGS CRAM** | Patient_4 | 643.4 | 429 | 409.6 | 424 | 175.4 | 1072 | -72.7 | 1.2516 | 1.2516 |
| **hcWGS CRAM** | Patient_5 | 519.1 | 212 | 329.2 | 194 | 133.4 | 956 | -74.3 | -549 | -549 |
| **hcWGS CRAM** | Patient_6 | 446.6 | 316 | 305.6 | 314 | 115.2 | 924 | -74.2 | 0.6747 | 0.6747 |
| **hcWGS CRAM** | Patient_7 | 508.0 | 280 | 556.2 | 228 | 159.3 | 1058 | -68.6 | -271 | -271 |
| **hcWGS CRAM** | Patient_8 | 513.2 | 259 | 354.6 | 196 | 198.5 | 989 | -61.3 | 294 | 294 |
| **hcWGS CRAM** | avg. hcWGS CRAM | 588.1 | 265 | 371.1 | 258 | 149.6 | 982 | -74.6 |  |  |
| **lcWGS BAM** | Patient_10 | 26.6 | 6 | 15.7 | 91 | 7.9 | 585 | -70.4 | -1.5219 | -1.5219 |
| **lcWGS BAM** | Patient_11 | 22.4 | 94 | 11.8 | 11 | 9.8 | 585 | -56.3 | -1.7247 | -1.7247 |
| **lcWGS BAM** | Patient_12 | 35.0 | 11 | 18.1 | 18 | 11.4 | 587 | -67.5 | 197 | 197 |
| **lcWGS BAM** | Patient_13 | 38.4 | 93 | 28.3 | 9 | 17.1 | 588 | -55.4 | -0.0995 | -0.1044 |
| **lcWGS BAM** | Patient_14 | 50.8 | 13 | 26.0 | 72 | 15.4 | 587 | -69.8 | 0.6685 | 0.6685 |
| **lcWGS BAM** | Patient_15 | 29.4 | 8 | 25.8 | 92 | 13.1 | 587 | -55.5 | -0.3753 | -0.3753 |
| **lcWGS BAM** | Patient_16 | 22.3 | 19 | 13.4 | 10 | 9.3 | 585 | -58.4 | -1.1042 | -1.1042 |
| **lcWGS BAM** | Patient_17 | 21.9 | 11 | 19.2 | 21 | 7.6 | 594 | -65.2 | 0.8085 | 0.8085 |
| **lcWGS BAM** | Patient_18 | 24.4 | 14 | 18.1 | 92 | 5.7 | 586 | -76.5 | -0.7351 | -0.7351 |
| **lcWGS BAM** | Patient_19 | 20.1 | 94 | 11.5 | 93 | 6.3 | 586 | -68.5 | 2.246 | 2.246 |
| **lcWGS BAM** | Patient_20 | 30.9 | 10 | 17.3 | 21 | 9.2 | 585 | -70.1 | -0.8571 | -0.8571 |
| **lcWGS BAM** | Patient_21 | 32.9 | 10 | 18.8 | 9 | 9.0 | 585 | -72.8 | -1.1035 | -1.1035 |
| **lcWGS BAM** | Patient_22 | 18.8 | 70 | 10.1 | 92 | 7.8 | 585 | -58.4 | -0.6158 | -0.6158 |
| **lcWGS BAM** | Patient_23 | 34.2 | 92 | 20.4 | 78 | 8.3 | 586 | -75.6 | -0.3781 | -0.3781 |
| **lcWGS BAM** | Patient_24 | 36.9 | 11 | 28.2 | 92 | 10.8 | 589 | -70.9 | -0.4761 | -0.5149 |
| **lcWGS BAM** | Patient_25 | 27.7 | 13 | 20.5 | 93 | 8.0 | 586 | -71.2 | -0.2155 | -0.2155 |
| **lcWGS BAM** | Patient_26 | 16.5 | 11 | 9.0 | 94 | 6.4 | 584 | -61.0 | 1.6129 | 1.6129 |
| **lcWGS BAM** | Patient_27 | 43.1 | 9 | 26.0 | 9 | 12.8 | 588 | -70.3 | -0.8908 | -0.8908 |
| **lcWGS BAM** | Patient_28 | 47.6 | 13 | 35.1 | 91 | 21.7 | 588 | -54.4 | 0.0057 | 0.0057 |
| **lcWGS BAM** | Patient_9 | 23.6 | 9 | 16.9 | 79 | 6.5 | 589 | -72.6 | 759 | 759 |
| **lcWGS BAM** | avg. lcWGS BAM | 30.2 | 31 | 19.5 | 58 | 10.2 | 587 | -66.2 |  |  |
| **lcWGS CRAM** | Patient_10 | 20.3 | 122 | 12.2 | 11 | 8.7 | 608 | -57.0 | -1.5219 | -1.5219 |
| **lcWGS CRAM** | Patient_11 | 24.2 | 120 | 12.4 | 10 | 7.2 | 567 | -70.1 | -1.7247 | -1.7247 |
| **lcWGS CRAM** | Patient_12 | 68.2 | 123 | 28.6 | 154 | 11.2 | 615 | -83.6 | 197 | 197 |
| **lcWGS CRAM** | Patient_13 | 61.3 | 122 | 30.9 | 21 | 12.2 | 619 | -80.1 | -0.0995 | -0.1044 |
| **lcWGS CRAM** | Patient_14 | 53.0 | 14 | 27.8 | 157 | 14.4 | 610 | -72.8 | 0.6685 | 0.6685 |
| **lcWGS CRAM** | Patient_15 | 33.2 | 22 | 18.5 | 121 | 9.3 | 578 | -72.0 | -0.3753 | -0.3753 |
| **lcWGS CRAM** | Patient_16 | 23.7 | 101 | 13.8 | 14 | 8.2 | 556 | -65.6 | -1.1042 | -1.1042 |
| **lcWGS CRAM** | Patient_17 | 23.8 | 12 | 12.6 | 184 | 7.7 | 583 | -67.7 | 0.8085 | 0.8085 |
| **lcWGS CRAM** | Patient_18 | 24.7 | 120 | 19.2 | 9 | 7.7 | 603 | -68.9 | -0.7351 | -0.7351 |
| **lcWGS CRAM** | Patient_19 | 21.4 | 12 | 11.9 | 7 | 6.7 | 599 | -68.6 | 2.246 | 2.246 |
| **lcWGS CRAM** | Patient_20 | 32.7 | 102 | 13.2 | 14 | 10.3 | 590 | -68.4 | -0.8571 | -0.8571 |
| **lcWGS CRAM** | Patient_21 | 22.5 | 120 | 17.7 | 149 | 10.0 | 585 | -55.6 | -1.1035 | -1.1035 |
| **lcWGS CRAM** | Patient_22 | 28.5 | 104 | 11.0 | 15 | 5.9 | 543 | -79.4 | -0.6158 | -0.6158 |
| **lcWGS CRAM** | Patient_23 | 31.9 | 104 | 14.8 | 100 | 6.9 | 547 | -78.4 | -0.3781 | -0.3781 |
| **lcWGS CRAM** | Patient_24 | 70.0 | 123 | 37.2 | 156 | 14.7 | 614 | -79.0 | -0.4761 | -0.5149 |
| **lcWGS CRAM** | Patient_25 | 41.9 | 11 | 16.0 | 12 | 11.8 | 544 | -71.8 | -0.2155 | -0.2155 |
| **lcWGS CRAM** | Patient_26 | 18.3 | 102 | 9.0 | 11 | 8.4 | 591 | -53.8 | 1.6129 | 1.6129 |
| **lcWGS CRAM** | Patient_27 | 47.9 | 12 | 29.6 | 11 | 13.1 | 575 | -72.7 | -0.8908 | -0.8908 |
| **lcWGS CRAM** | Patient_28 | 60.1 | 123 | 25.6 | 11 | 21.5 | 571 | -64.3 | 0.0057 | 0.0057 |
| **lcWGS CRAM** | Patient_9 | 23.8 | 120 | 11.8 | 11 | 10.8 | 606 | -54.7 | 759 | 759 |
| **lcWGS CRAM** | avg. lcWGS CRAM | 36.6 | 84 | 18.7 | 59 | 10.3 | 585 | -71.7 |  |  |

**Supplementary Table 1:** Runtime, memory and telomere content estimation comparison between TelomereHunter2 version 1.0.0. and TelomereHunter version 1.1. Reported for single instances and group averages for low-coverage (~4x) and high-coverage (~141x) WGS samples in BAM and CRAM format. The tel_content equals GC-corrected, log2 transformed tumor versus control estimated telomere content.


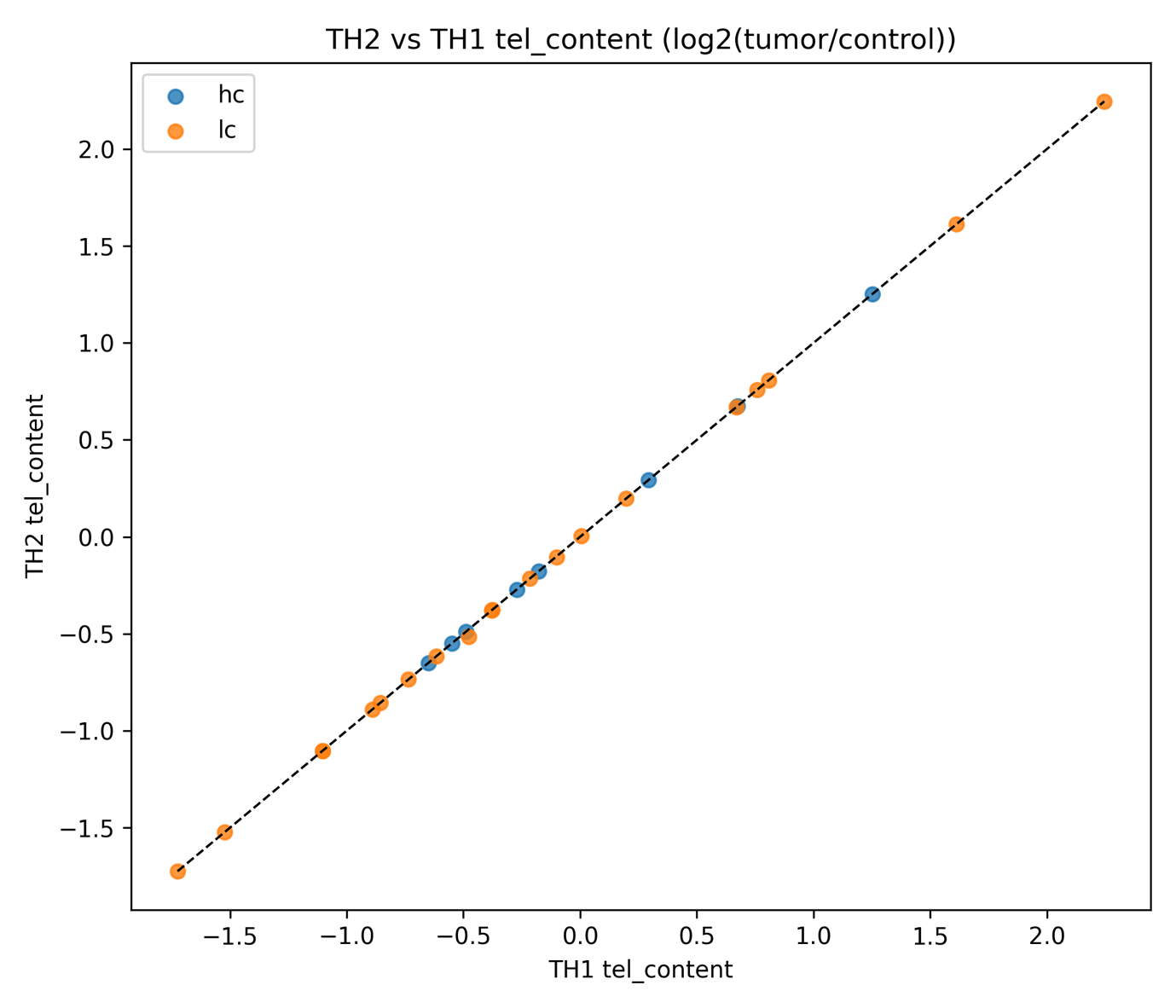


**Supplementary Figure 1:** Correlation of tel_content (log2(Tumor/Control)) between TelomereHunter2 version 1.0.0. and TelomereHunter version 1.1 for all samples from the low-coverage (lc) and high-coverage (hc) WGS samples. Pearson r=1.000 (p=4.63e-56), Spearman r=0.999 (p=5.01e-40).

**Supplementary Figure 2:** **Comparative Telomere Content Across Dog Breeds**
Boxplot illustrating telomere content across four dog breeds: Bulldog (n = 4), Golden Retriever (n = 5), Labrador Retriever (n = 6), and Mastiff (n = 7). Median telomere content is marked by the central black line within each box, while the boxes represent interquartile ranges (IQRs). Data was obtained from (Meadows et al., 2023). The reference genome was “UU_Cfam_GSD_1.0_ROSY“.

**Supplementary Figure 3:** **Comparative Telomere Content Across Mouse Breeds**
Boxplot illustrating telomere content across two mouse breeds: *Mus musculus musculus* (n = 16) and *Mus musculus domesticus* (n = 19). Median telomere content is marked by the central black line within each box, while the boxes represent interquartile ranges (IQRs). Data was obtained from (Harr et al., 2016). The reference genome was “GRCm39/mm39”.

**Supplementary Figure 4:** **Single-cell ATAC Analysis for Telomere Content across Cell types**
scATAC data were obtained from the 10x Genomics public dataset "10k Human PBMCs, ATAC v2, Chromium Controller" (10x Genomics, 2022), annotated with exemplary cell types from snapATACv2 analysis (Zhang et al., 2024) documented in the repository. TelomereHunter2 was used to extract telomere content from the bam files with minimum reads per cell barcode of 30,000. A: Number of cells per cell type and their share of cells with telomeric reads B: Telomere content per single cell grouped by cell type.

## Sources

10x Genomics. (2022). *10k Human PBMCs, ATAC v2, Chromium Controller, Single Cell ATAC Dataset by Cell Ranger ATAC 2.1.0* [Dataset].

Harr, B., Karakoc, E., Neme, R., Teschke, M., Pfeifle, C., Pezer, Ž., Babiker, H., Linnenbrink, M., Montero, I., Scavetta, R., Abai, M. R., Molins, M. P., Schlegel, M., Ulrich, R. G., Altmüller, J., Franitza, M., Büntge, A., Künzel, S., & Tautz, D. (2016). Genomic resources for wild populations of the house mouse, Mus musculus and its close relative Mus spretus. *Scientific Data*, *3*(1), 160075. https://doi.org/10.1038/sdata.2016.75

Meadows, J. R. S., Kidd, J. M., Wang, G.-D., Parker, H. G., Schall, P. Z., Bianchi, M., Christmas, M. J., Bougiouri, K., Buckley, R. M., Hitte, C., Nguyen, A. K., Wang, C., Jagannathan, V., Niskanen, J. E., Frantz, L. A. F., Arumilli, M., Hundi, S., Lindblad-Toh, K., Ginja, C., … Ostrander, E. A. (2023). Genome sequencing of 2000 canids by the Dog10K consortium advances the understanding of demography, genome function and architecture. *Genome Biology*, *24*(1), 187. https://doi.org/10.1186/s13059-023-03023-7

Zhang, K., Zemke, N. R., Armand, E. J., & Ren, B. (2024). A fast, scalable and versatile tool for analysis of single-cell omics data. *Nature Methods*, *21*(2), 217–227. https://doi.org/10.1038/s41592-023-02139-9
